# Cryptochrome 2 acetylation attenuates its antiproliferative effect in breast cancer

**DOI:** 10.1038/s41419-023-05762-8

**Published:** 2023-04-06

**Authors:** Kangkai Xia, Shujing Li, Yuxi Yang, Xiaoxia Shi, Binggong Zhao, Linlin Lv, Zhiqiang Xin, Jie Kang, Ping Ren, Huijian Wu

**Affiliations:** 1 School of Bioengineering & Key Laboratory of Protein Modification and Disease, Liaoning Province, Dalian University of Technology, Dalian, 116024 China; 2 The Second Hospital of Dalian Medical University, Dalian, 116024 China

**Keywords:** Acetylation, Breast cancer

## Abstract

Breast cancer is the most commonly diagnosed cancer, and its global impact is increasing. Its onset and progression are influenced by multiple cues, one of which is the disruption of the internal circadian clock. *Cryptochrome 2 (Cry2)* genetic dysregulation may lead to the development of some diseases and even tumors. In addition, post-translational modifications can alter the Cry2 function. Here, we aimed to elucidate the post-translational regulations of Cry2 and its role in breast cancer pathogenesis. We identified p300-drived acetylation as a novel Cry2 post-translational modification, which histone deacetylase 6 (HDAC6) could reverse. Furthermore, we found that Cry2 inhibits breast cancer proliferation, but its acetylation impairs this effect. Finally, bioinformatics analysis revealed that genes repressed by Cry2 in breast cancer were mainly enriched in the NF-κB pathway, and acetylation reversed this repression. Collectively, these results indicate a novel Cry2 regulation mechanism and provide a rationale for its role in breast tumorigenesis.

## Introduction

Cryptochrome 2 (Cry2) is a component of the negative limb of the circadian transcription-translation feedback loop [1, 2]. In mammals, *Cry1/2* and *Per1/2/3* transcription is activated by a heterodimer of circadian locomoter output cycles protein kaput (CLOCK) and brain and muscle ARNT-like 1 (BMAL1) by binding to the E-box elements in their promoters [3]. Cry1/2 and Per1/2/3 translocate to the nucleus and inhibit their transcription by repressing the CLOCK/BMAL1 heterodimer for a period of about 24 h [2, 4]. In addition to transcription regulation, the ubiquitin ligases F-box and leucine-rich repeat protein 3 and F-box and leucine-rich repeat protein 21 mediate the ubiquitin-mediated proteasomal degradation of Cry1/2 [5, 6], while ubiquitin specific peptidase 7 removes Cry1/2 ubiquitination [7]. Furthermore, Cry1/2 are phosphorylated by dual specificity tyrosine phosphorylation regulated kinase 1a and glycogen synthase kinase 3 beta for better recognition and ubiquitination by F-box and leucine-rich repeat protein 3 [8, 9].

Apart from their roles in the circadian clock, Cry1 and Cry2 play key roles in a variety of physiological pathways. Additionally, Cry1 and Cry2 mutations alter tumor formation [10]. However, when exploring circadian clock proteins functions in tumor formation and progression, most studies investigated Cry1/2 simultaneously, assuming that their roles were the same and even redundant. Surprisingly, their functions are divergent or even opposite, both outside the circadian rhythm. For example, in response to DNA damage, *Cry2*-deficient primary fibroblasts exhibit reduced *p21* transcription, whereas loss of *Cry1* promotes *p21* expression [11]. Only Cry2 stimulates the degradation of the critical tumor activator MYC by recruiting it to SCF^FBXL3^, whereas Cry1 does not [12]. This suggests that Cry2 may play a more dominant role than Cry1 in altering cancer-related signaling pathways. Overall, the roles of Cry1 and Cry2 in regulating tumor formation or progression are distinct and unique; therefore, their potential functions in tumor development must be examined separately. A systematical analysis of genomic profiling and clinical data showed significantly decreased Cry1 and Cry2 mRNA levels in multiple cancer types, including breast carcinoma, lung squamous cell carcinoma, thyroid carcinoma, and lung adenocarcinoma [13]. Altered Cry2 expression, however, is more likely to be associated with altered activity of established oncogenic or tumor suppressor pathways in breast cancer than Cry1 [13]. The global impact of breast cancer, a common type of tumor, is increasing. With 2.3 million new cases and 680 thousand new deaths reported in 2020, breast cancer has become the most commonly diagnosed cancer worldwide [14]. The incidence of breast cancer is much higher in the developed world, which suggests that aspects of modern lifestyle may influence its progression. One possibility involves a circadian clock disorder [15]. However, in addition to circadian rhythm disorders due to environmental time cues, alterations in the function of circadian proteins themselves can also affect breast cancer development through non-circadian rhythm pathways. The link between tumorigenesis and these circadian proteins suggests that manipulating them might be a remedial approach for treating cancer.

The alteration in specific protein function could be caused not only by altered abundance due to upstream gene dysregulation, but also by the precise regulation of post-translational modifications. Among the diverse modification types, reversible acetylation provides an elegant mechanism to govern protein function and is involved in many cellular processes, such as the cell cycle, DNA damage repair, transcriptional regulation, and cell metabolism [16]. Many proteins in the circadian molecular clock are regulated by acetylation. For instance, TIP60 can acetylated BMAL1 to recruit bromodomain containing 4 and P-TEFb, followed by productive elongation of circadian transcripts [17]. In addition to the canonical acetyltransferase, CLOCK acts as a non-canonical acetyltransferase that can acetylate BMAL1, thereby weakening the inhibitory effect of Cry1 on the CLOCK/BMAL1 transcription complex [18]. Moreover, silent mating-type information regulator 2 homolog 1 (SIRT1) deacetylated Per2 and enhanced its degradation [19].

SIRT7 was found to remove Cry1 acetylation in mice [20], promoting its degradation and regulating hepatic glucose homeostasis. Therefore, we investigated whether homologous Cry2 could be acetylated and whether its acetylation plays a specific role in breast cancer progression. We found that Cry2 could be acetylated by p300 and removed by HDAC6. Furthermore, Cry2 inhibited breast cancer development; however, Cry2 acetylation reversed this effect, demonstrating that circadian proteins and their post-translational modifications affect breast cancer progression.

## Results

### Cry2 is a substrate of acetylation driven by p300

**Fig. 1:**
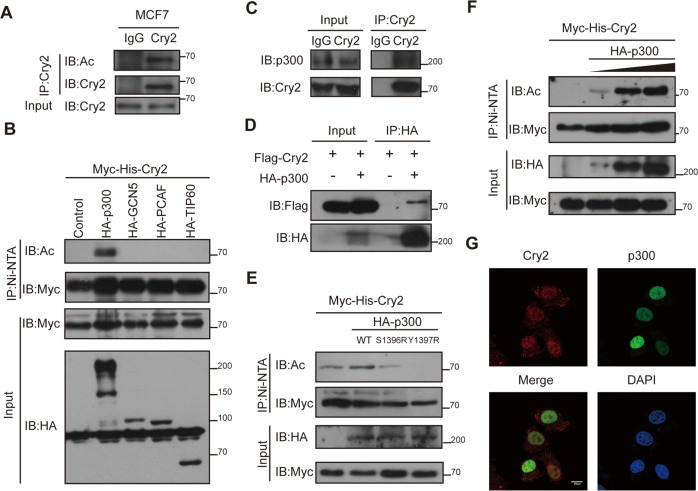
Cry2 is a substrate of acetylation which is driven by p300. **A** Endogenous Cry2 is targeted by acetylation in MCF7 cells. **B** p300 promoted Cry2 acetylation in HEK293T cells. **C** Endogenous interaction between Cry2 and p300 in MCF7 cells. **D** Exogenous interaction between Cry2 and p300 in HEK293T cells. **E** p300 promoted Cry2 acetylation depend on its KAT activity. **F** p300 promoted Cry2 acetylation in a dose-dependent manner. **G** Cry2 and p300 were colocalized in the nucleus in MCF7 cells.

A previous study reported Cry1 acetylation, while SIRT7 could deacetylate Cry1 [20]. Although Cry1 and Cry2 are homologs, there are some different sequences in the C-terminal domain, where the Cry1 acetylation sites are located [21, 22] (Fig. S1); therefore, it is necessary to test whether Cry2 is also acetylated. Acetylation of endogenous Cry2 in lysates from MCF7 and T47D breast cancer cell lines, were detected using an anti-acetylated lysine antibody following of Cry2 immunoprecipitation (Figs. 1A and S2). Acetylation is generated by the lysine acetyltransferase (KAT)-catalyzed transfer of an acetyl group to lysine, and 90% of non-histone proteins are acetylated by a series of canonical KATs, including p300, TIP60, GCN5, and PCAF [16]. To identify the upstream KAT responsible for Cry2 acetylation, Cry2 was co-expressed with these four KATs separately. We found that only p300 significantly induced Cry2 acetylation, suggesting that this may be the involved KAT (Fig. 1B). Later, interactions were detected between Cry2 and p300 at the endogenous level in MCF7 cells and at the exogenous level in 293T cells via co-immunoprecipitation (Fig. 1C, D). To examine whether the intrinsic KAT activity of p300 is involved in Cry2 acetylation, we co-expressed Cry2 with WT p300 and two catalytically inactive p300 mutants (S1396R and Y1397R). Cry2 acetylation was induced by WT p300 expression but not by the mutants (Fig. 1E) [23, 24], and co-transfection with p300 increased Cry2 acetylation in a dose-dependent manner (Fig. 1F). Thus, we conclude that p300 KAT activity is required for Cry2 acetylation. At the same time, immunofluorescence analysis demonstrated that Cry2 co-localized with p300 mainly in the nucleus in MCF7 cells (Fig. 1G). These data indicate that Cry2 is subjected to acetylation through the KAT p300.

### HDAC6 is the Cry2 deacetylase

**Fig. 2:**
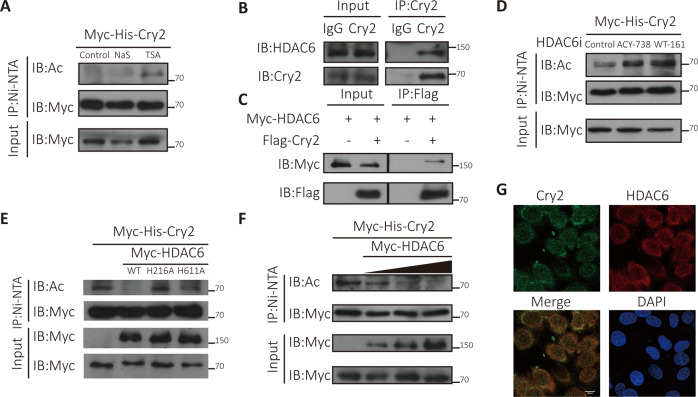
The KDAC of Cry2 is HDAC6. **A** HDACs inhibitors increased Cry2 acetylation. **B** Endogenous interaction between Cry2 and HDAC6 in MCF7 cells. **C** Exogenous interaction between Cry2 and HDAC6 in HEK293T cells. **D** HDAC6 inhibitors increased Cry2 acetylation. **E** HDAC6 inhibited Cry2 acetylation depend on its KDAC activity. **F** HDAC6 decreased Cry2 acetylation in a dose-dependent manner. **G** Cry2 and HDAC6 were colocalized in the cytoplasm in MCF7 cells.

Reversible acetylation is catalyzed by KATs and reversed by lysine deacetylases, grouped into two categories: classical zinc-dependent HDACs and NAD^+^-dependent SIRTs [16]. In the study of Cry1 acetylation, the acetylation of exogenous Cry2 was not altered in the presence of nicotinamide (NAM), a SIRTs inhibitor, indicating that the lysine deacetylase of Cry2 was not a member of the SIRTs family; therefore, we focused on the HDACs family. Cells were treated with two pan-inhibitors of HDACs, sodium butyrate (NaS) and trichostatin (TSA), to observe the change in Cry2 acetylation level. TSA significantly increased the Cry2 acetylation level, but NaS did not (Fig. 2A). The difference between these two inhibitors is that TSA can inhibit HDAC6 enzymatic activity but NaS cannot [25, 26]; thus, our results indicate that HDAC6 might be involved in Cry2 deacetylation. In addition, an endogenous interaction between Cry2 and HDAC6 was found in MCF7 cells using an anti-Cry2 antibody via co-immunoprecipitation (Fig. 2B). In 293 T cells, co-immunoprecipitation was used to demonstrate the exogenous interaction between Cry2 and HDAC6 (Fig. 2C). Treatment with two selective HDAC6 inhibitors, ACY-738 and WT-161, decreased Cry2 acetylation, implying that HDAC6 likely triggered Cry2 deacetylation (Fig. 2D). Indeed, HDAC6, but not deacetylase-inactive mutants (H216A and H611A), efficiently removed Cry2 acetylation (Fig. 2E) [27]. Furthermore, an increase in HDAC6 decreased Cry2 acetylation (Fig. 2F). Thus, we concluded that HDAC6 deacetylase activity is required for Cry2 deacetylation. Immunofluorescence analysis also showed that Cry2 and HDAC6 co-localized in the cytoplasm (Fig. 2G). Taken together, our data showed that HDAC6 can deacetylate Cry2.

### Cry2 is acetylated at K560, K576, and K591

**Fig. 3:**
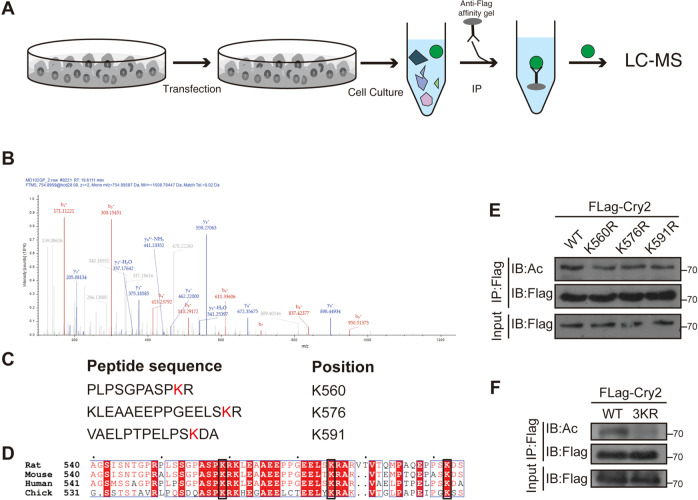
K560/576/591 are the acetylation sites of Cry2. **A** Schematic diagram of the process of identifying Cry2 acetylation sites. **B** MS analysis of the Cry2-derived peptides containing acetylated K591. **C** Mass spectrometry analysis identification of the Cry2-derived peptides containing potential acetylated sites. **D** The potential acetylation sites of Cry2 are conserved in multiple species. **E** Monomutation of the potential acetylated lysine reduced the acetylation level of Cry2. **F** The acetylation of Cry2–3KR was abolished.

Given that Cry2 can be acetylated by p300 and deacetylated by HDAC6, identifying its acetylation sites is essential. Flag-Cry2 was purified from 293 T cells and subjected to liquid chromatography-tandem mass spectrometry (Fig. 3A). This analysis identified Lys560, Lys576, and Lys591 as potential Cry2 acetylation sites in cells (Figs. 3B, C and S3). These three lysine residues within the Cry2 C-terminus are conserved in Cry2 orthologs and differ from those in Cry1 (Fig. 3D). Thus, three lysine residues were individually mutated to arginine residues, which mimicked the deacetylated state of Cry2. As shown in Fig. 3E, the three mutants showed decreased acetylation compared to that of Cry2-WT (Fig. 3E). Furthermore, we generated Flag-Cry2–3KR, wherein K560, K576, and K591 were all mutated to R, whereas acetylation of the Cry2 3KR mutant was not detected (Fig. 3F). Taken together, these data suggest that Cry2 was acetylated at K560, K576, and K590.

### Acetylation leads to Cry2 stabilization

**Fig. 4:**
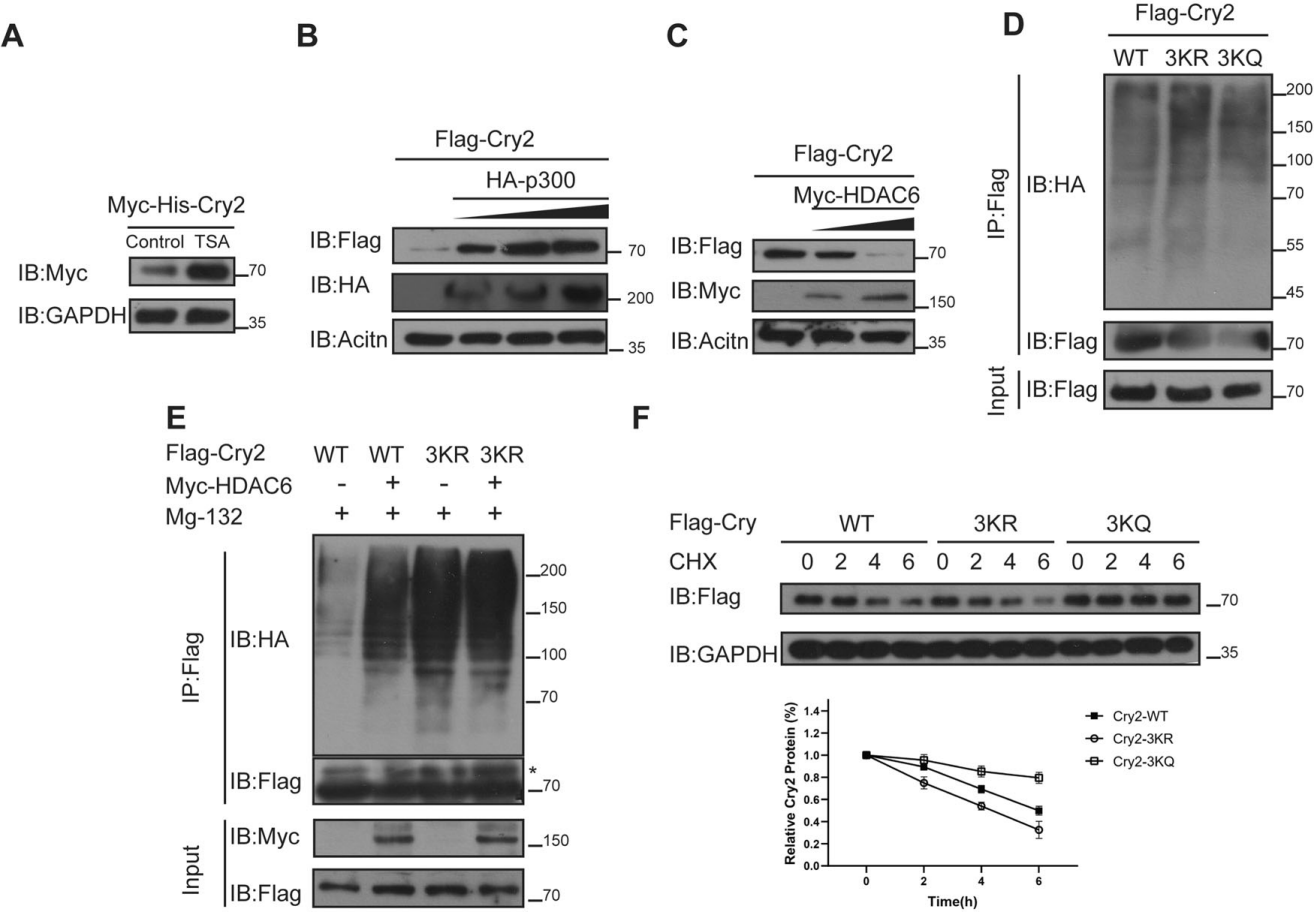
Acetylation leads to Cry2 stabilization. **A** TSA promoted Cry2 stability. **B** p300 promoted Cry2 stability in a dose-dependent manner. **C** HDAC6 impaired Cry2 stability in a dose-dependent manner. **D** Cry2–3KR increased poly-ubiquitination of Cry2. **E** HDAC6 could not alter the ubiquitination of Cry2–3KR. **F** Half-lives of Cry2 and mutants.

An increase in Cry2 protein level was observed when 293 T cells were treated with TSA (Fig. 4A). To determine whether acetylation alters Cry2 stabilization, p300 was overexpressed in a gradient, and Cry2 abundance gradually increased in a dose-dependent manner with p300 expression (Fig. 4B). In contrast, Cry2 protein levels gradually decreased with increasing HDAC6 expression (Fig. 4C). Lysine acetylation can regulate protein degradation through ubiquitination-proteasome-dependent pathways. Consequently, we hypothesized that acetylation regulates Cry2 degradation via the ubiquitination-proteasome pathway. The results showed that the polyubiquitination level of Cry2–3KR was significantly higher than that of Cry2-WT, but Cry2–3KQ, which mimics the acetylation state of lysine, abolished this increase (Fig. 4D). Moreover, when Cry2 was co-expressed with HDAC6, the poly-ubiquitination level of Cry2-WT increased, but the presence or absence of HDAC6 had no effect on Cry2–3KR (Fig. 4E). This suggests that Cry2 acetylation regulated its protein stability by escaping from polyubiquitination-mediated proteasomal degradation. Next, to determine the effect of Cry2 acetylation on its half-life, we expressed Cry2-WT, Cry2–3KR, and Cry2–3KQ in 293 T cells and performed cycloheximide chase experiments. The data revealed that Cry2 half-life was prolonged in Cry2–3KQ, but shortened in Cry2–3KR (Fig. 4F). In short, Cry2 acetylation resulted in reduced ubiquitination and increased stabilization of the Cry2 protein.

Cry2 is translated in the cytoplasm and exerts a repressive transcriptional function in the nucleus [2]. Meanwhile, p300 is mainly located in the nucleus, whereas HDAC6 is mainly located in the cytoplasm, and Cry2 can interact with both separately. Therefore, we examined whether acetylation changes the subcellular localization of Cry2. Immunofluorescence staining of Cry2 in MCF7 cells showed no difference in its nuclear localization in the absence or presence of HDAC6 inhibitors (Fig. S4); therefore, we concluded that acetylation of Cry2 did not affect its subcellular distribution.

### Cry2 acetylation in breast cancer cells impairs its ability to inhibit cell proliferation

**Fig. 5:**
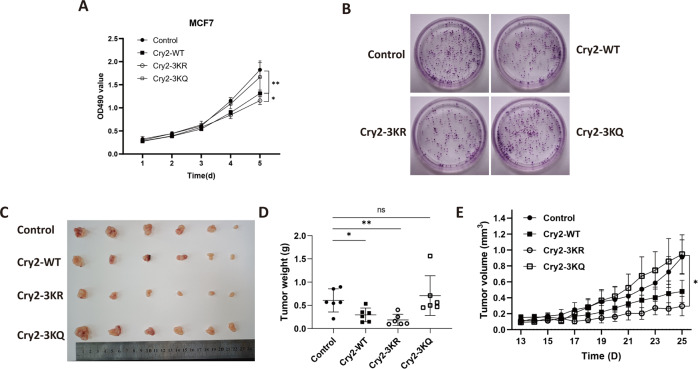
Acetylation of Cry2 in breast cancer cells impairs its ability to inhibit cell proliferation. **A** Cell viability was analyzed by CCK8. Means ± SD, *n* = 5. *p < 0.05; **p < 0.01. **B** Cell growth was determined by Colony formation. **C** Xenograft tumors from MCF7 cells. **D**, **E** Weight and growth of tumors in (**C**). Means ± SD, *n* = 6. *p < 0.05; **p < 0.01; ns, no significance.

Cry2 expression is altered in various cancer types, especially in breast cancer [28]. Cry2 knockdown reportedly increased the expression of several genes involved in cell proliferation [29]; therefore, we hypothesized that Cry2 and its reversible acetylation are involved in regulating breast cancer cell proliferation. To test this effect, CCK8 experiments were performed in MCF7 cells overexpressing Cry2-WT and Cry2–3KR, which could not be acetylated, as well as Cry2–3KQ, which mimics the acetylation state of lysine. Cry2-WT overexpression in MCF7 cells caused a significant decrease in proliferation rate, compared with the control (Fig. 5A), which is consistent with previous studies. At the same time, cells expressing Cry2–3KR exhibited reduced proliferation compared to control cells. Similar results were observed in T47D cells (Fig. S5). Moreover, cells expressing Cry2–3KQ showed no reduction in proliferation. Colony formation revealed an apparent decrease in colony formation when Cry2-WT was overexpressed compared to the control cells (Fig. 5B). Cells overexpressing Cry2–3KQ displayed more vigorous growth and had larger colonies than those overexpressing Cry2–3KR. MCF7 cells stably transfected with Cry2-WT, Cry2–3KR, Cry2–3KQ, or the control vector were inoculated into nude mice in a xenograft assay to test the tumor-suppressing role of Cry2. All mice injected with MCF7 cells developed tumors within 4 weeks. Compared with the control group, cells expressing Cry2-WT formed smaller tumors at a slower rate (Fig. 5C–E). As expected, cells that expressed Cry2–3KR displayed minor tumors and the slowest tumor growth rate. These results strongly indicated that Cry2 suppressed breast cancer cell proliferation, but acetylation impaired its inhibitory effect.

### Cry2 suppresses breast cancer cell proliferation through the NF-κB pathway

**Fig. 6:**
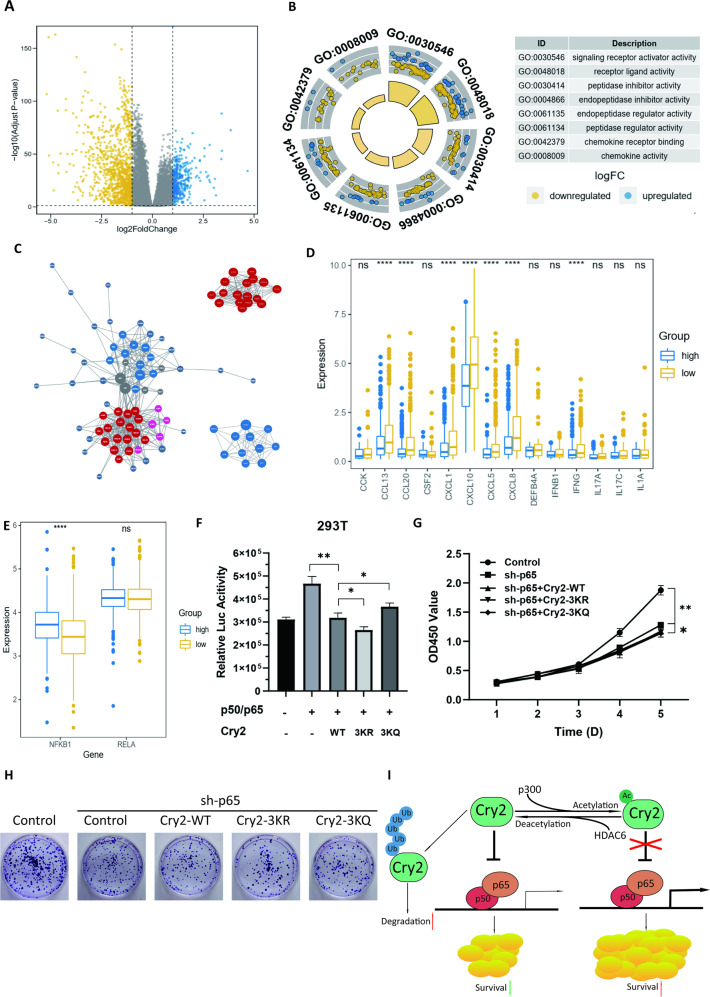
Cry2 suppresses breast cancer cell proliferation through the NF-ΚB pathway. **A** Volcano plot of DEGs between Cry2^High^ and Cry2^Low^ in the TCGA-BRCA datasets. **B** GO_MF analysis of Cry2 correlated genes in TCGA-BRCA. **C** PPI networks of DEGs of signaling receptor activator activity in (**B**). The first and second hub genes with nodes of higher degree shown in red and blue. **D** Genes expression in Cry2^High^ (*n* = 532) and Cry2^Low^ (*n* = 532) group. The genes are the red group shown in (**C**), *p < 0.05; ****p < 0.0001. **E** *RelA*(p65) and *NFKB1*(p50) expression in Cry2^High^ (*n* = 532) and Cry2^Low^ (*n* = 532) group. *p < 0.05; ****p < 0.0001. **F** Cry2–3KR enhances and Cry2–3KQ impairs the inhibitory effect of Cry2-WT on NF-κB-Luc. Means ± SD, *n* = 3. *p < 0.05; **p < 0.01. **G** CCK8 assays showed Cry2 acetylation does not alter its proliferation-inhibiting effect after the knockdown of *p65* in MCF7 cells. Means ± SD, *n* = 3. *p < 0.05; **p < 0.01. **H** Colony formation assays showed Cry2 acetylation does not alter its proliferation-inhibiting effect after the knockdown of *p65* in MCF7 cells. **I** Proposed model depicting the regulation of Cry2 by acetylation and impact of Cry2 acetylation in breast cancer cell proliferation.

Considering that Cry2 mainly represses transcriptional activation in cells, we analyzed the TCGA-BRCA database to explore the possible pathways, through which Cry2 suppresses breast cancer cell proliferation. The sample of cancer cases in TCGA -BRCA cohort was split equally into two groups. Cases with Cry2 expression higher than the median were deemed as the “High” group, and the rest as the “Low” group. We analyzed differentially expressed genes (DEGs) between the Cry2^High^ and Cry2^Low^ groups in the dataset. Fold change >2 and adjusted *p*-value < 0.05 were used as the cutoffs for DEGs. Most DEGs were downregulated (Fig. 6A). This transcription repression could be explained by the high level of Cry2 expression. GO analysis was used to explore the functions of these DEGs. Regarding molecular function, signaling receptor activator activity was the most significantly altered molecular function, which contained 78 DEGs, most of which were downregulated (Fig. 6B, Table 1). Therefore, the proteins translated from these enriched DEGs were subjected to protein-protein interaction analysis, and four groups of significant associations were identified using MCODE analysis (Fig. 6C). At the same time, previous studies demonstrated that Cry2 might repress transcription activators, such as hypoxia inducible factor 1 subunit alpha and glucocorticoid receptor. Therefore, these 78 DEGs were analyzed for their upstream transcriptional activators. They were mainly regulated by the NF-κB pathway, whose transcription complex is p65/p50. Moreover, the cluster containing the most proteins in MCODE analysis was also regulated by the NF-κB pathway (Fig. 6C). Therefore, we analyzed the genes in cluster 1 (Red in Fig. 6C) and found that the expression of related genes was suppressed in the Cry2^High^ group, compared with the Cry2^Low^ group (Fig. 6D). In contrast, the *p65* mRNA level was unchanged, and the *p50* mRNA level was upregulated (Fig. 6E). Therefore, the expression of related downstream genes may be due to the high expression of Cry2. A luciferase reporter gene assay was used to examine the effect of Cry2 on NF-κB transcriptional activity. As shown, Cry2-WT obviously repressed the transcriptional activity of the p65/p50 complex, but Cry2–3KQ almost lost this inhibition (Fig. 6F and Fig. S6). Considering the above analysis, Cry2 could inhibit the NF-κB pathway, whereas acetylation of Cry2 could weaken or even eliminate this inhibition. NF-κB promotion of cell proliferation via autophagy inhibition has been reported, and we also found that Cry2 could inhibit breast cancer proliferation. Therefore, we speculated that Cry2 may inhibit proliferation by inhibiting the NF-κB pathway. CCK8 and colony formation assays were performed in *p65*-knockdown MCF7 cells overexpressing Cry2-WT, Cry2–3KR, and Cry2–3KQ. As shown in Fig. 6G, H, we found that *p65* deletion abrogated the effect of Cry2 acetylation on breast cancer cell proliferation. In conclusion, Cry2 inhibits breast cancer cell proliferation via the NF-κB pathway.

**Table 1:** The 78 genes analyzed for Fig. 6C.

CXCL5	CCL20	GAL	CCL7	CXCL1
CHGB	CXCL10	ANGPT4	FAM3D	OGN
CCL18	FGF21	CCL8	FGF14	THBS4
CXCL8	CCL14	IL36RN	SEMA6D	GDF10
MIA	CARTPT	INSL6	IGF1	NRTN
GDF5	CCL16	SLURP1	IL1A	NTS
CALCB	RLN2	DKK1	GHRH	CRLF1
IL36G	EPHA7	LEP	CCL13	GREM2
CXCL11	IFNG	IL17C	WNT6	POMC
ADIPOQ	CXCL6	PTN	PENK	LACRT
IL17B	STC2	EDN3	FGF10	GAST
CALCA	CCK	CSF2	IFNL1	CMTM5
RETNLB	IFNB1	EPO	CSH2	INSL4
PF4V1	REG1A	IL36B	PPBP	IAPP
SST	IFNL2	GH2	FGF23	C10orf99
IL17A	DEFB4A	IL36A		

## Discussion

Although an increasing number of studies have indicated a relationship between altered clock genes and breast cancer risk [30–32], few studies have focused on the regulation of post-translational modifications of circadian proteins in breast cancer progression. We have previously reported that CLOCK SUMOylation stimulates breast cancer cell growth by upregulating the transcriptional activity of estrogen receptor-alpha [33]. Herein, we present new evidence that post-translational modifications of circadian proteins regulate breast cancer progression. Cry2 suppressed breast cancer proliferation through the NF-κB pathway, but acetylation of Cry2 attenuated this effect (Fig. 6I).

In previous studies, *Cry2* showed a significant decrease in breast cancer [13], and higher *Cry2* expression was associated with a longer metastasis-free survival time [29], suggesting that this circadian protein may be a tumor suppressor in breast cancer. In our study, Cry2 expression inhibited breast cancer cell proliferation, and acetylation of Cry2 reversed this effect (Fig. 5). Cry2 acetylation by p300 and deacetylation by HDAC6 were identified in breast cancer cells. Low Cry2 acetylation level showed a stronger inhibition of breast cancer cell growth, which inhibited by HDAC6, consistent with a previous report showing that high HDAC6 expression was associated with higher survival in breast cancer patients [34]. Moreover, when p300, the Cry2 KAT, was inhibited, the tumor burden of breast cancer was reduced in a murine model [35]. This is consistent with our finding that high Cry2 acetylation level promotes breast cancer proliferation. This study supports the effects of HDAC6 and p300 in breast cancer; however, we should also recognize that the role of specific KATs in breast cancer might involve the synthetic action of its acetylated substrate. A previous study reported that the e74-like factor 5 acetylation mediated by p300 is vital for e74-like factor 5 inhibition of breast cancer cells proliferation [24]. This result contradicts our findings in this paper that Cry2 acts as a tumor suppressor in the low acetylation state, and e74-like factor 5 acts as a tumor suppressor in the high acetylation state; interestingly, both acetylation reactions are mediated by p300. Taken together, the substrate proteins should be examined more closely when studying the effects of acetylation on the occurrence and development of breast cancer.

TNFα significantly increased in *Cry2* knockdown cells, and a lack of Cry2 protein could activate pro-inflammatory cytokine expression [36, 37]. TNFα and NF-κB pathway activate each other in positive feedback loop. In our bioinformatics analysis, Cry2 inhibited the transcription of several downstream genes of the NF-κB pathway (Fig. 6D); therefore, we speculate that Cry2 might inhibit TNFα expression by suppressing the transcriptional activation of the p65/p50 complex. Several lines of evidence support the regulation of NF-κB activity by circadian proteins. First, CLOCK recruitment by p65 at the NF-κB-response element was shown to enhance NF-κB target genes [38]. Second, BMAL1 enhances p65 acetylation and further activates the NF-κB signaling pathway [39]. Third, RAR related orphan receptor A, another component of the circadian pathway, upregulates IκBα expression, thereby inhibiting NF-κB activity [40]. These studies support the mutual regulation between circadian proteins and the NF-κB pathway, and we hypothesized that Cry2 is a negative regulator of the NF-κB pathway. However, how Cry2 inhibits the NF-κB pathway and how acetylated Cry2 impairs this inhibitory effect remain unknown. Cry2, as a transcription repressor, directly binds to and inhibits multiple transcription activators, including hypoxia inducible factor 1 alpha, a member of the basic helix-loop-helix family comprising CLOCK and BMAL1, as well as some nuclear receptors, including the glucocorticoid receptor, pregnane X receptor and constitutive androstane receptor [5, 41, 42]. The p65/p50 transcription factor complex is crucial in the NF-κB pathway [43, 44]. Therefore, it could be hypothesized that Cry2 might also directly bind to and inhibit the p65/p50 complex, thus inhibiting the NF-κB pathway. In addition, the mechanism, by which Cry2 acetylation weakens this inhibitory effect, requires further investigation.

Cry2 stability increased under p300 expression and decreased under HDAC6 expression (Fig. 4B, C). These results demonstrate crosstalk between Cry2 acetylation and ubiquitination. The reason for ubiquitination changes in Cry2 after acetylation is worth exploring. Lysine acetylation enhances Cry1 stability by blocking ubiquitination of the same residue [20]. However, the ubiquitinated-lysine residues of Cry2, including K125/241/347/474/503, do not compete directly with the acetylated- lysine residues K560/576/591 [45, 46]. Therefore, we believe that the change in ubiquitination caused by acetylation is not due to competition between two modifications of the same lysine. Moreover, the phosphorylated-serine residues, including Ser-554 and Ser-558, are in close proximity to acetylated-lysine residues [8, 9]. We speculated that the decreased ubiquitination of acetylated -Cry2 might be due to its reduced of Cry2 phosphorylation, which remains a matter for further discussion. As a circadian protein, the stability of Cry2 plays a critical role in circadian rhythm period, which would be extended by a null mutation of Cry2 [5, 47]. Cry2 acetylation prolongs its half-life (Fig. 4F), which may shorten the circadian rhythm period, possibly leading to uncoupling of the external light cycle and intrinsic circadian rhythm. A regular and robust circadian rhythm is associated with slow-growing tumors in the breast, whereas irregular rhythms are associated with faster tumor growth [48]. Therefore, it is worth investigating whether the Cry2 acetylation status affects the progression of breast cancer by altering the circadian rhythm of breast cancer cells.

In summary, this study revealed the effect of Cry2 itself and its acetylation on breast cancer cell proliferation and identified the regulatory enzymes of Cry2 acetylation. This once again demonstrates that circadian proteins and their post-translational modifications may influence breast cancer development through non-circadian pathways and provides a new idea for the mechanism of breast cancer formation and progression.

## Materials and Methods

### Plasmids and antibodies

Flag-Cry2 and Myc-His-Cry2 were kindly provided from Dr. YingHui Fu (University of California, San Francisco, USA). Myc-HDAC6 was provided from Dr. Jiadong Wang (Peking University Health Science Center, Beijing). HA-p300/PCAF/GCN5/TIP60, HA-Ub, NF-κB-luc, EGFP-p65, Flag-p50, and sh-p65 used in our previous studies [24, 39]. Anti-Cry2 Ab (Proteintech, 13997-1-AP, Chicago, USA; Abcam, ab 155255, Cambridge, UK),anti-p300 Ab (Beyotime,AF6795, Shanghai,China), anti-HDAC6 Ab (Beyotime, AF7071, Shanghai,China), anti-HA Ab (GeneTex, GTX115044, San Antonio, TX, USA), anti-Flag Ab (Sigma-Aldrich, F7425, Saint Louis, Mo, USA), anti-Myc Ab (Origene, TA150121, Rockville, MD, USA), anti-acetylated-lysine Ab (Cell Signaling Technology, #9441, Boston, MA, USA), anti-β-actin Ab (Santa Cruz Biotechnology, sc-9104, CA, USA), anti-GAPDH Ab (GeneTex, GTX627408, CA, USA).

### Cell culture

Cell lines MCF7, T47D, HEK293T, and COS7 were purchased from China Infrastructure of Cell Line Resource for our previous experiments. The MCF7 cells were cultured in Eagle’s Minimum Essential Medium supplemented with 0.01 mg/ml of human recombinant insulin and 10% FBS. The T47D cells were cultured in RPMI-1640 supplemented with 0.01 mg/ml of bovine insulin and 10% FBS. Dulbecco’s modified Eagle’s medium supplemented with 10% FBS was used to culture HEK293T and COS7.

### Chemicals

The MG132 was purchased from Selleck (Selleck, Houston, TX, USA) and used at final concentrations of 200μM. NaS (Aladdin, Shanghai, China). Trichostatin A (TSA) and cycloheximide (CHX) were purchased from Sigma-Aldrich (Sigma-Aldrich, Saint Louis, Mo, USA) and used at a final concentration of 2μM, 50μM. ACY-738 and WT-161 were obtained from MCE and used at a final concentration of 2.5μM and 1μM.

### Western blot and immunoprecipitation

Cells were lysed with TNE lysis buffer (20 mM Tris-HCl pH 7.4, 100 mM NaCl, 1 mM EDTA, 0.5% NP-40, 10% glycerol and complete protease inhibitor). The standard protocol for Western blot analysis was followed. Briefly, samples were separated in SDS-PAGE gels and transferred to polyvinylidene difluoride membranes (Millipore, Boston, MA, USA). 5% milk was used to block the membranes and the antibodies were immunoblotted with them. The bands were visualized using chemiluminescence. (Advansta, San Jose, CA, USA).

Immunoprecipitations were performed by incubating cells extract with the relevant antibody overnight at 4 °C, and then adding 60ul Protein A/G mix magnetic beads for another 2 h at 4 °C. A SDS-PAGE analysis and immunoblot with the indicated antibodies were performed after the immunoprecipitated complexes had been washed three times with TNE lysis buffer.

### Immunofluorescence

1 × 10^4^ MCF7 cells were seeded on glass coverslips, fixed with 4% paraformaldehyde for 15 min at room temperature, and incubated for 5 min with 100 mM glycine/PBS. Cells were then blocked with 1% bovine serum albumin and incubated overnight at 4 °C with antibodies. After washing three times with phosphate-buffered saline, cells were incubated for one hour at 4 °C with secondary antibody. After washing out the unbound antibodies, the cells were stained for 30 min with DAPI. As a final step, fluorescence microscopy was used to obtain microscopic images.

### Luciferase reporter assay

COS7 or 293 T cells were plated in 24-well plates. The cells were transfected with 100 ng NF-κB-Luc reporter plasmids and other plasmids required in each group. 24 h after transfection, cells were detected using dual-luciferase reporter assay kit (Promega, Madison, WI, USA) according to the manufacturer’s instructions.

### LC-MS/MS analysis

293 T cells were transfected with Flag-Cry2 and p300 for 48 h, and then were lysed with TNE buffer. Flag-Cry2 was purified by anti-Flag Affinity Gel and subjected to SDS-PAGE. After Coomassie blue staining, the bands containing Flag-Cry2 were excised from the gel and collected. The protein was reduced with acetonitrile and dithiothreitol. The peptides were digested with trypsin at 37 °C overnight. The peptides were dissolved with liquid chromatography mobile phase A and then separated using an EASY-nLC 1000 ultra-high performance liquid phase system. The resulting MS/MS data were processed using Proteome Discoverer 2.1.

### Cell proliferation assay

1 × 10^3^ transfected cells were plated in 96-well plates, and the cell proliferation assay was performed with Cell Counting Kit-8 (Bimake, Houston, USA). In colony formation assays, plasmid-transfected cells were plated into 6-well plates and stained with crystal violet once clones were clearly visible.

### Xenograft model

All experiments were carried out according to the regulation set by the Ethics Committee for Biology and Medical Science of Dalian University of Technology. Female BALB/C nude mice were obtained from Liaoning Changsheng Biotechnology. About 1 × 10^6^ transfected MCF7 cells were suspended in 80 μl of serum-free medium, and mixed with Matrigel (BD, USA) at 1:1 ratio then the mixture was injected subcutaneously into the left flank of the nude mice. The tumor volume was determined using the formula L × W^2^ × 0.5, where L is the longest diameter and W is the shortest diameter.

### Statistical analysis

Data were presented as mean ± SDs and Student’s t-test (unpaired, two-tailed) was used to compare two groups of independent samples. All the experiments were repeated at least three times. Statistical significance was considered at the *p* < 0.05 level.

### TCGA data

The RNA-seq data of TCGA were downloaded from UCSC XENA (https://xenabrowser.net/datapages/). Gene ontology (GO) analysis was performed using EnrichGO function in the “clusterProfiler” R package.

## Supplementary information

Supplementary Figure

Original Data File

aj-checklist

## Data Availability

The data that support the findings of this study are available from the corresponding author upon request.
