# Supplementary material for: Cryptochrome 2 acetylation attenuates its antiproliferative effect in breast cancer
Source: Cell Death Dis. 2023 Apr 6;14(4):250. doi: 10.1038/s41419-023-05762-8 (PMC10079955; doi:10.1038/s41419-023-05762-8)
Supplement: Supplementary file 1 — Supplementary Figure [file 41419_2023_5762_MOESM1_ESM.docx]

**Supplementary Figure**

**Figure S1** Cry1 and Cry2 are different in C-timernal. The black boxes are the acetylated-lysine residues of Cry1

**Figure S2** Endogenous Cry2 is targeted by acetylation in T47D cells.

**Figure S3** MS analysis of the Cry2-derived peptides containing acetylated K576(up) and K560(down).

**Figure S4** Cry2 localization in MCF7 cells is unchanged treated with HDAC6 inhibitors

**Figure S5** Cry2-WT and Cry2-3KR inhibits T47D cells proliferation. Growth curves of T47D cells were measured by CCK8 assay. Means ± SD, n=5. *p < 0.05; **p < 0.01.

**Figure S6** Cry2-3KR enhances and Cry2-3KQ impairs the inhibitory effect of Cry2-WT on NF-κB-Luc in COS7 cells. Means ± SD, n=3. *p < 0.05; **p < 0.01.

**
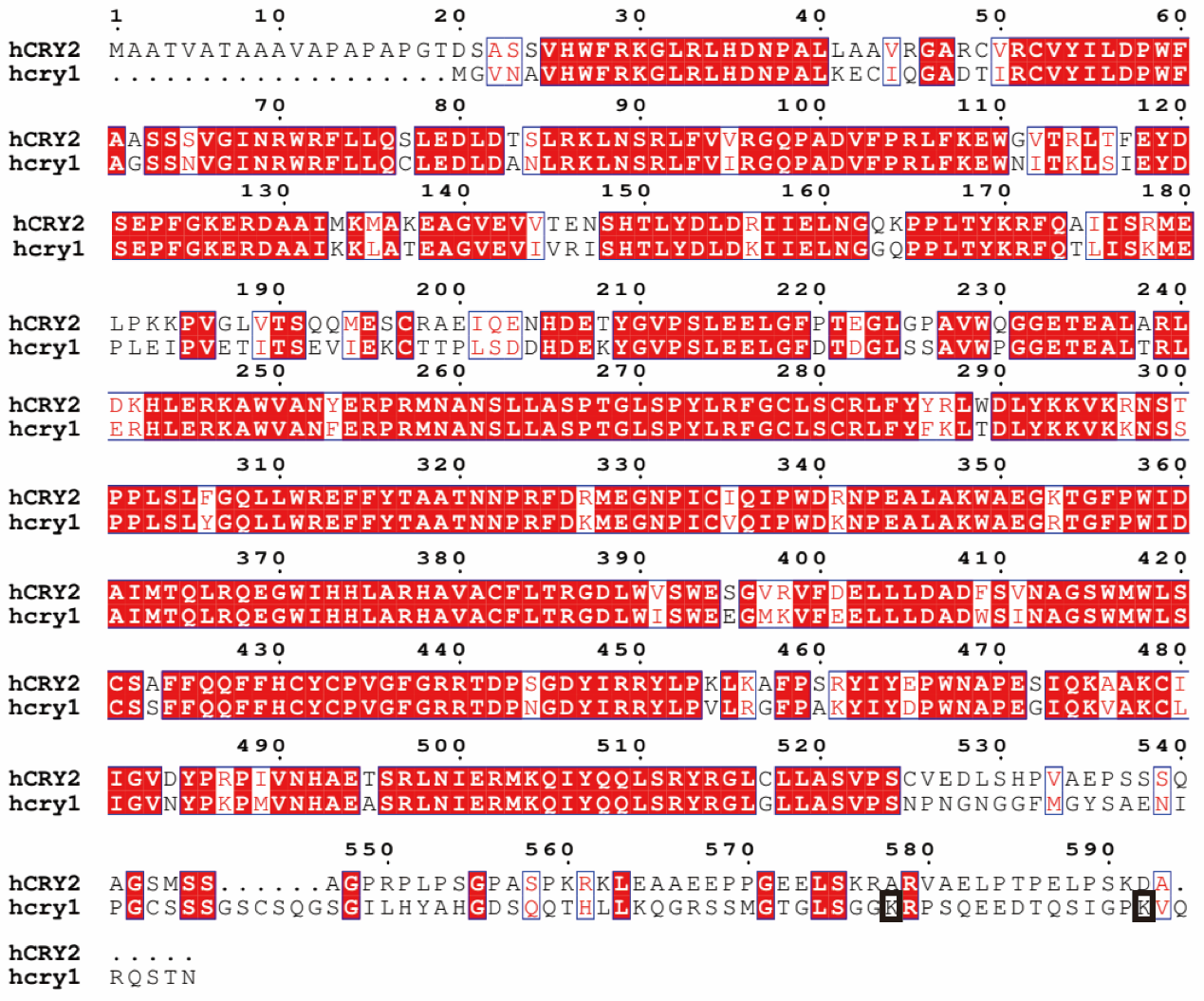
**

**Figure S1** Cry1 and Cry2 are different in C-timernal. The black boxes are the acetylated-lysine residues of Cry1

**
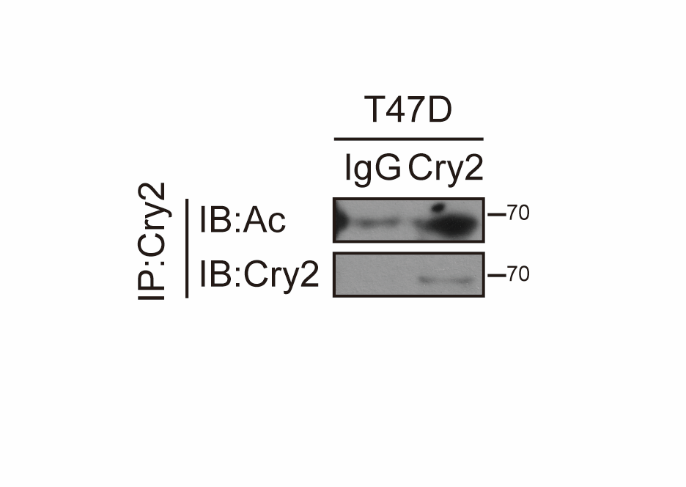
**

**Figure S2** Endogenous Cry2 is targeted by acetylation in T47D cells.

**
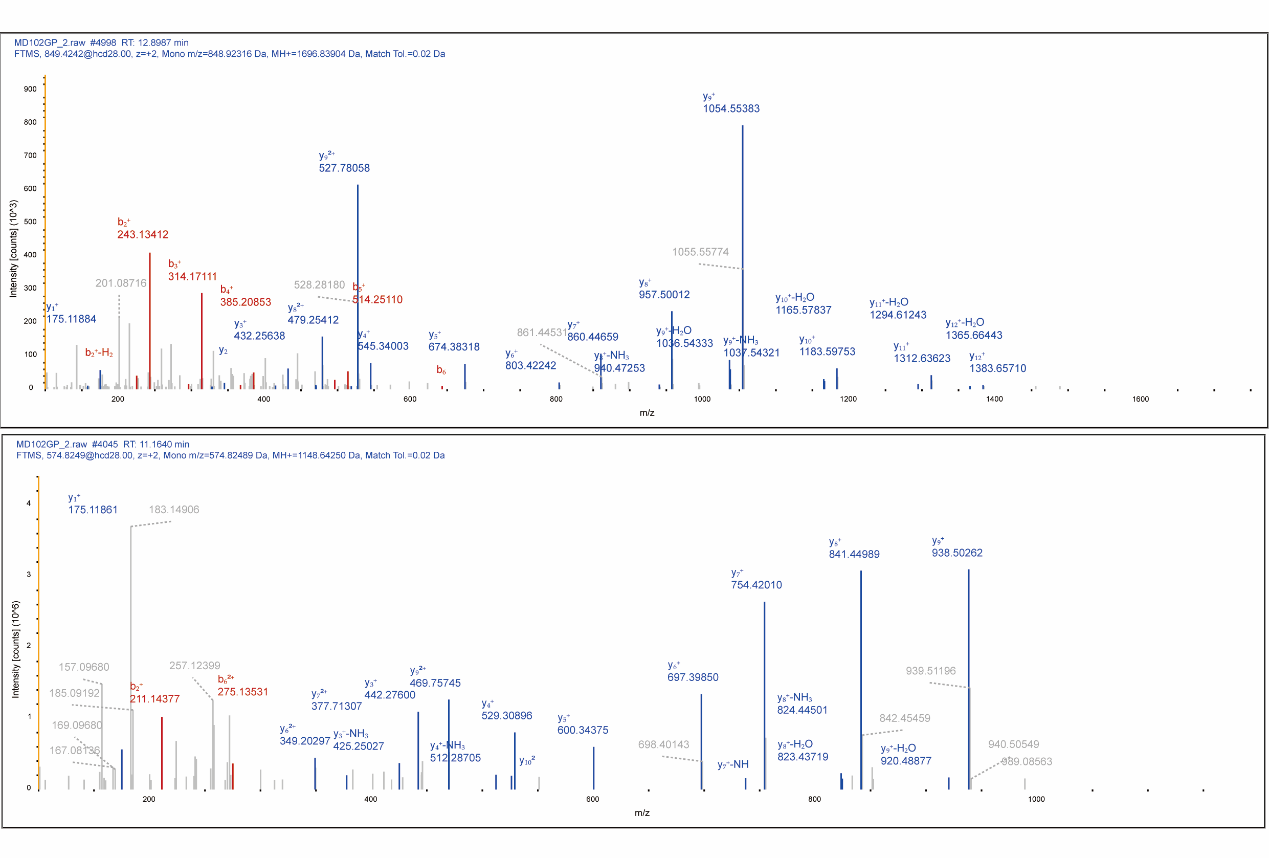
**

**Figure S3** MS analysis of the Cry2-derived peptides containing acetylated K576(up) and K560(down).

**
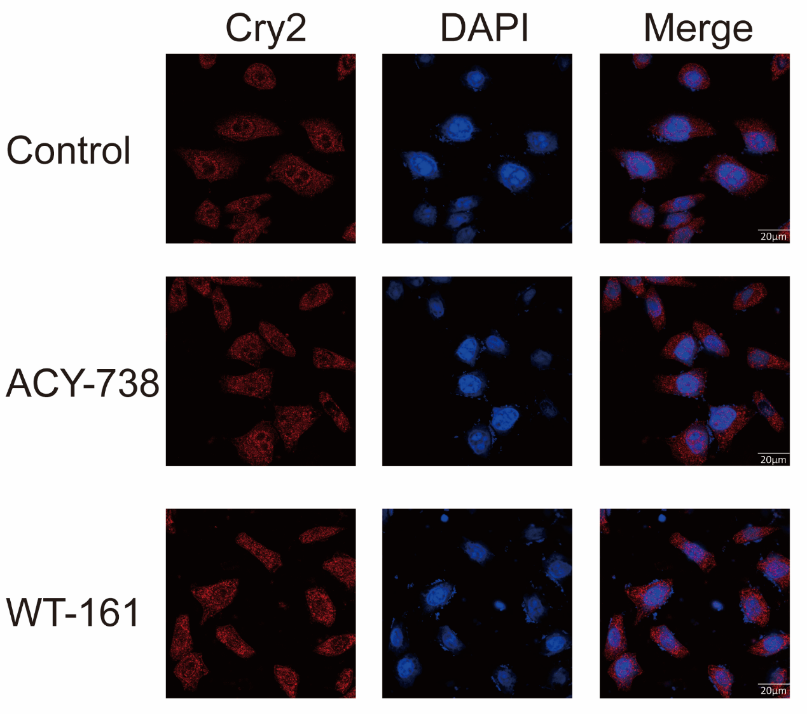
**

**Figure S4** Cry2 localization in MCF7 cells is unchanged treated with HDAC6 inhibitors

**
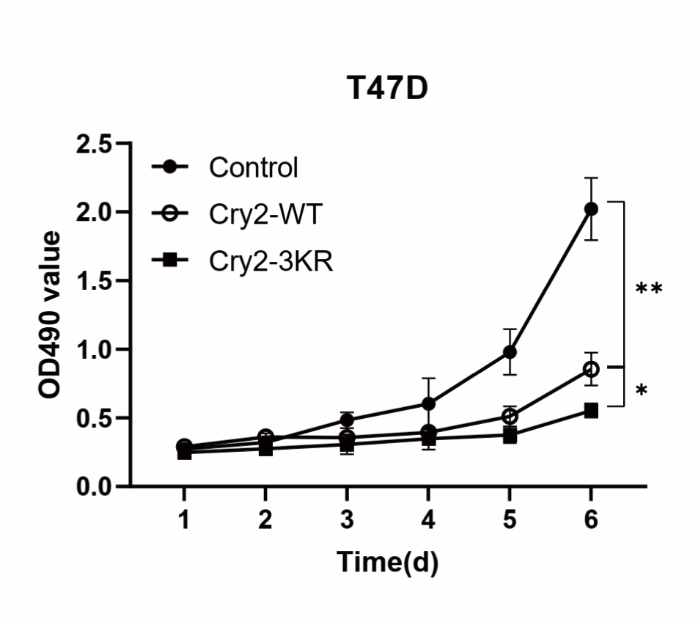
**

**Figure S5** Cry2-WT and Cry2-3KR inhibits T47D cells proliferation. Growth curves of T47D cells were measured by CCK8 assay. Means ± SD, n=5. *p < 0.05; **p < 0.01.**
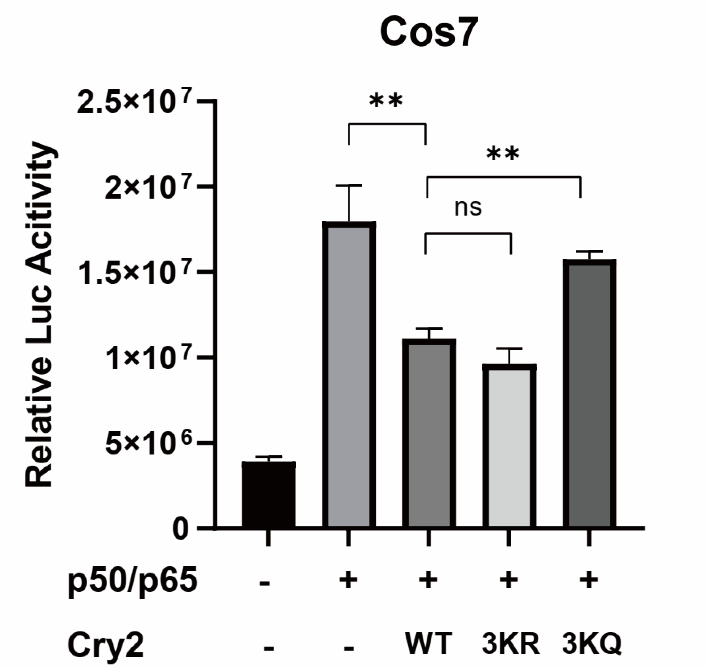
**

**Figure S6** Cry2-3KR enhances and Cry2-3KQ impairs the inhibitory effect of Cry2-WT on NF-κB-Luc in COS7 cells. Means ± SD, n=3. *p < 0.05; **p < 0.01.
