# Supplementary figures and images for: Cryptochrome 2 acetylation attenuates its antiproliferative effect in breast cancer
Source: Cell Death Dis. 2023 Apr 6;14(4):250. doi: 10.1038/s41419-023-05762-8 (PMC10079955; doi:10.1038/s41419-023-05762-8)

1A

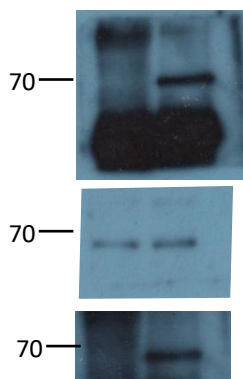

1D

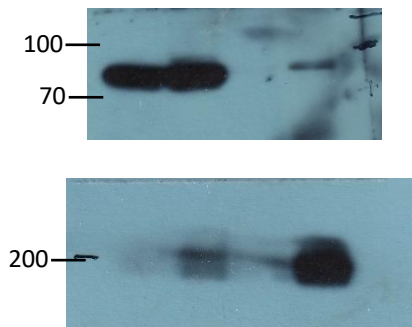

2A

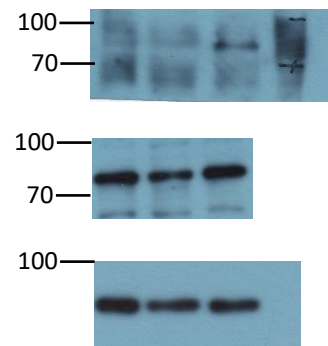

1B

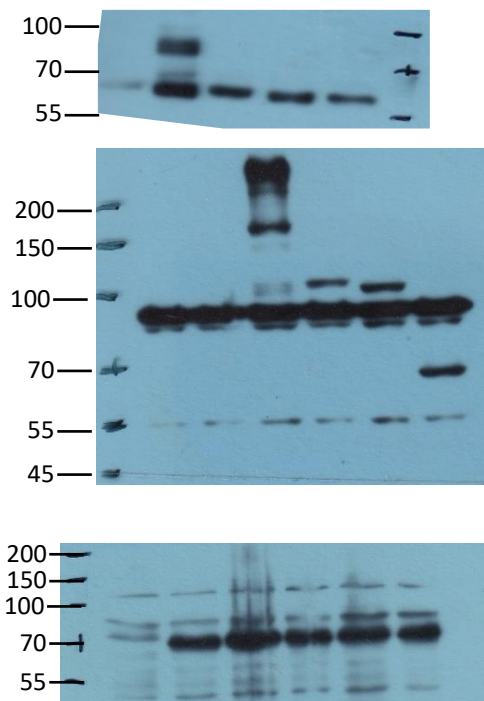

1E

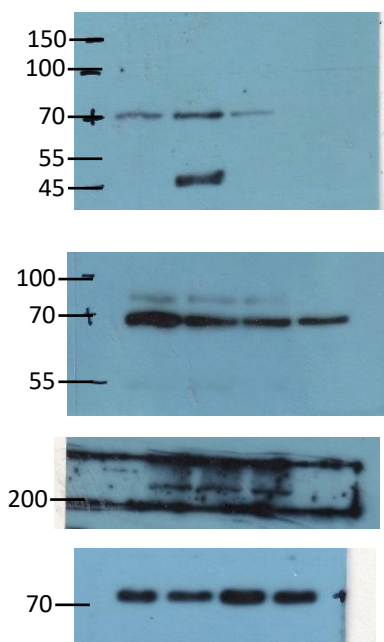

2B

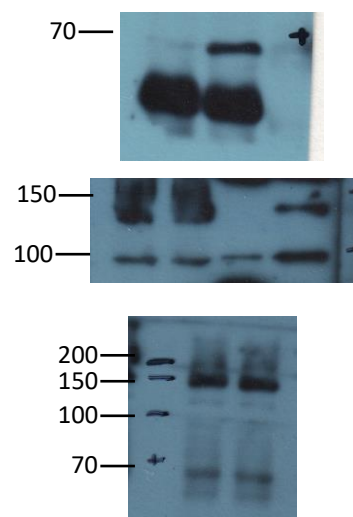

1C

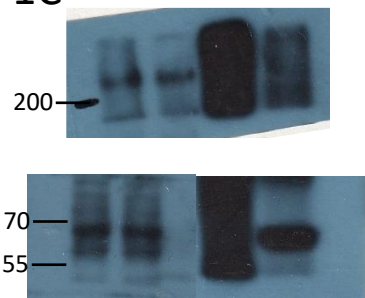

1F

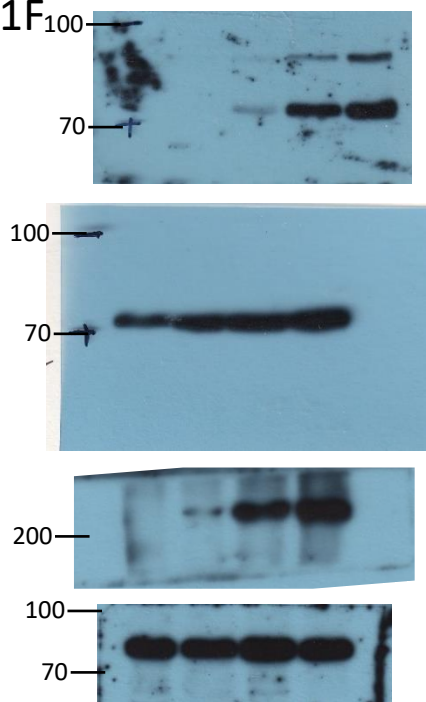

2C

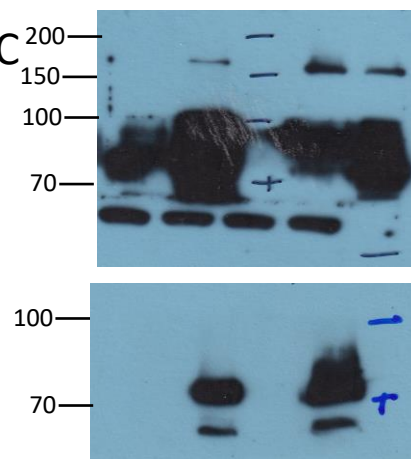

2D

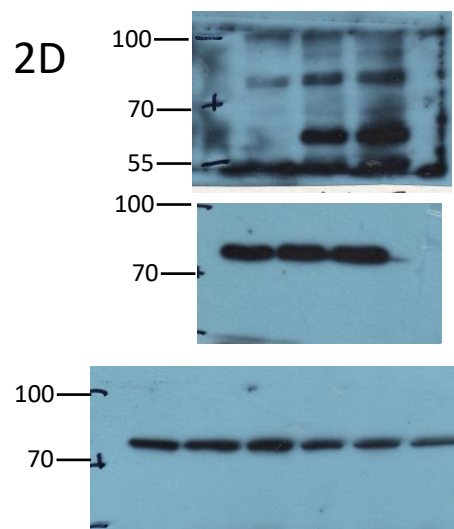

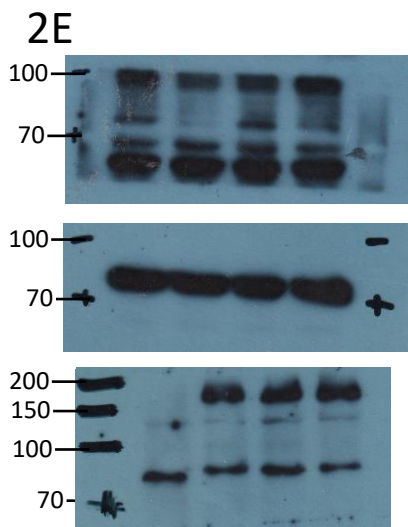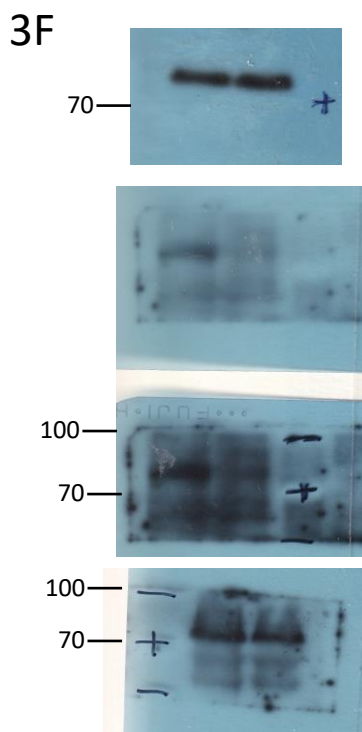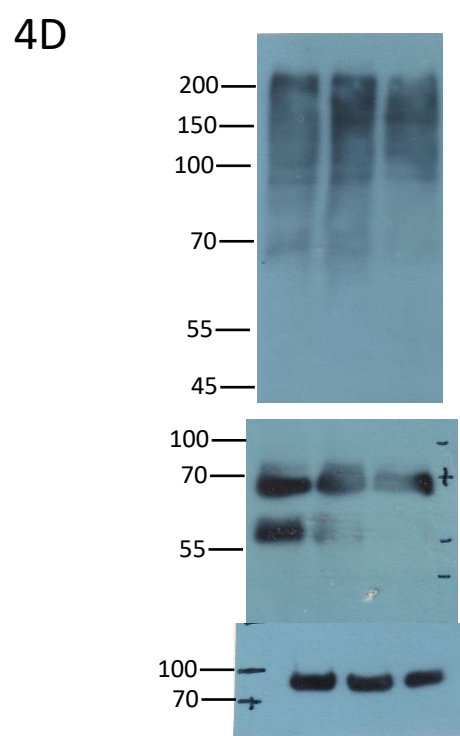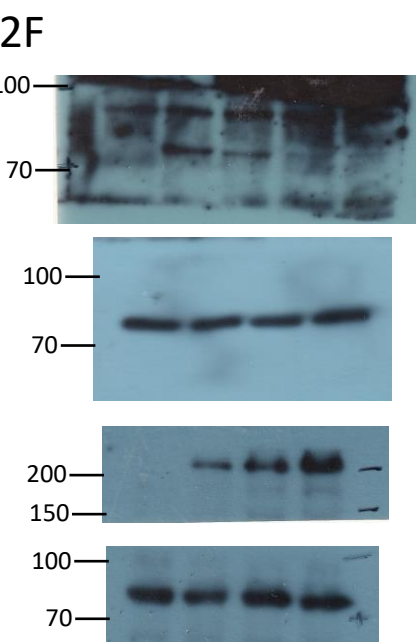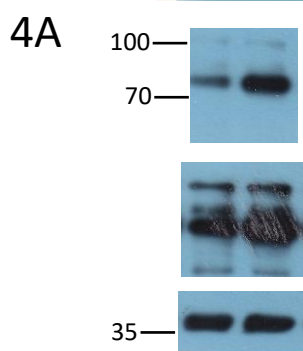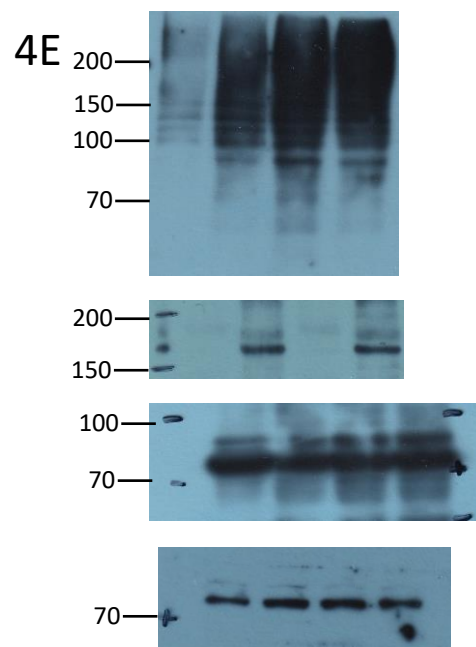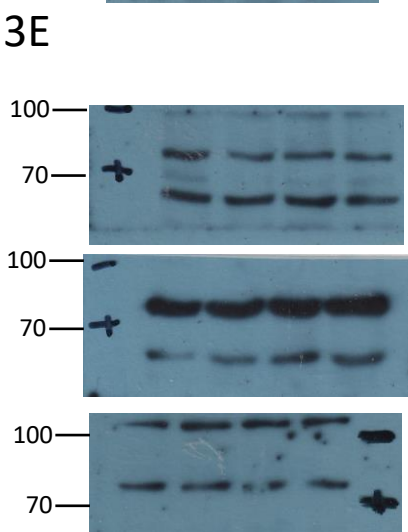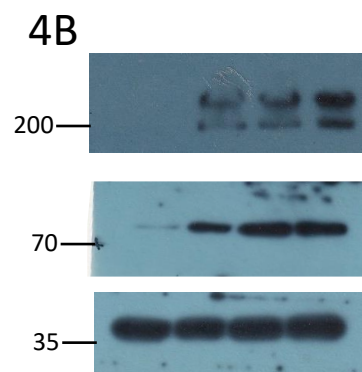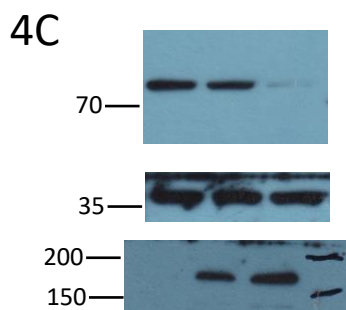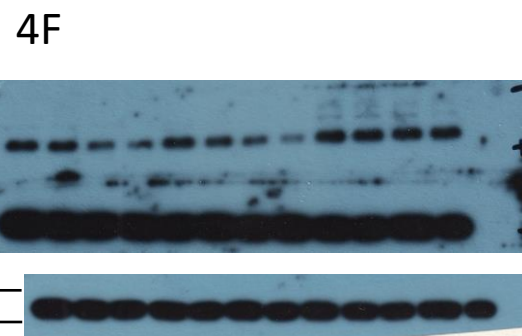

Supplement: Supplementary file 2 — Original Data File [file 41419_2023_5762_MOESM2_ESM.pdf]
